# Assessment of provider-initiated HIV screening in Nigeria with sub-Saharan African comparison

**DOI:** 10.1186/s12913-017-2132-4

**Published:** 2017-03-09

**Authors:** Felix A. Ogbo, Andrew Mogaji, Pascal Ogeleka, Kingsley E. Agho, John Idoko, Terver Zua Tule, Andrew Page

**Affiliations:** 1 Centre for Health Research, School of Medicine, Western Sydney University, Campbelltown Campus, Locked Bag 1797, Penrith, NSW 2571 Australia; 2 Departement of Psychology, Faculty of Social Science, Benue State University, PMB 102119, Makurdi, Nigeria; 3 Department of Public Health, School of Public Health, College of Science, Health and Engineering La Trobe University, Bundoora, VIC 3083, Australia; 4 School of Science and Health, Western Sydney University, Campbelltown Campus, Locked Bag 1797, Penrith, NSW 2571 Australia; 5 Department of Medicine, Faculty of Medical Sciences, University of Jos, P.M.B 2084, Jos, Plateau State Nigeria; 6 Prevention of Maternal-to-Child Transmission of HIV Unit, Benue State Ministry of Health, State Secretariat, High Level, PMB 102093, Makurdi, Benue State Nigeria; 7 General Hospital Idekpa, Ohimini Local Government Area, Benue State Hospitals Management Board, Makurdi, Benue State Nigeria

**Keywords:** Provider-initiated testing and counselling, HIV, AIDS, Benue State, Nigeria

## Abstract

**Background:**

Despite Nigeria’s high HIV prevalence, voluntary testing and counselling rates remain low. UNAIDS/WHO/CDC recommends provider-initiated testing and counselling (PITC) for HIV in settings with high HIV prevalence. We aimed to assess the acceptability and logistical feasibility of the PITC strategy among adolescents and adults in a secondary health care centre in Idekpa Benue state, Nigeria.

**Method:**

All patients (aged ≥ 13 years) who visited the out-patient department and antenatal care unit of General Hospital Idekpa, Benue state, Nigeria were offered PITC for HIV. The intervention was implemented by trained health professionals for the period spanning (June to December 2010).

**Results:**

Among the 212 patients who were offered PITC for HIV, 199 (94%) accepted HIV testing, 10 patients (4.7%) opted out and 3 patients (1.4%) were undecided. Of the 199 participants who were tested for HIV, 9% were HIV seropositive. The PITC strategy was highly acceptable and feasible, and increased the number of patients who tested for HIV by 5% compared to voluntary counselling and testing. Findings from this assessment were consistent with those from other sub-Saharan African countries (such as Uganda and South Africa).

**Conclusion:**

PITC for HIV was highly acceptable and logistically feasible, and resulted in an increased rate of HIV testing among patients. Public health initiatives (such as the PITC strategy) that facilitate early detection of HIV and referral for early treatment should be encouraged for broader HIV control and prevention in Nigerian communities.

## Background

Globally, sub-Saharan Africa remains the region most affected by the human immunodeficiency virus/acquired immunodeficiency virus (HIV/AIDS) [1]. In 2015, sub-Saharan Africa countries (including Nigeria) accounted for approximately 75% of new HIV infections in adults [2]. Despite the introduction of voluntary counselling and testing (VCT) for HIV in sub-Saharan Africa countries, more than half (60%) of people do not know their HIV status, and many living with HIV do not know that they are infected [3]. Consequently, majority of people living with HIV miss the opportunity for early detection and timely treatment, and also play an important role in the spread of the virus [4]. Of the annual incidence of HIV (approximately 2.6 million per year) worldwide [2], an estimated 40–90% of these cases experience symptoms of acute HIV infection, and a substantial number would seek medical care [5–7]. However, symptoms of acute HIV infection are often missed by primary care clinicians because these symptoms are similar to those of influenza and other viral illnesses [8].

To increase the number of people who test for HIV, the Joint United Nations Programme on HIV/AIDS/World Health Organization (UNAIDS/WHO, including the Centre for Disease Control, CDC) recommends provider-initiated testing and counselling (PITC) strategy for all patients aged ≥ 13 years. PITC refers to a routine offer of HIV testing and counselling for all patients who visit a health facility by a health professional (particularly in HIV endemic communities), regardless of the presenting symptoms, and without a separate written consent [9]. This intervention presents an important opportunity for preventive health care services in patients – a major tenet of primary care [10, 11]. The PITC strategy has been employed successfully in developing countries with high HIV prevalence. For example, studies from Uganda [12], Zambia [13] and South Africa [14] found that PITC strategy (opt-out) increased the proportion of patients who tested for HIV and the number of patients who commenced anti-retroviral therapy. These studies also showed that PITC was highly acceptable and feasible in the health facility. Similarly, a study from Nigeria conducted among a cohort of university students found that the opt-out strategy was feasible and highly acceptable [15].

In 2007, the Federal Ministry of Health in Nigeria adopted the PITC strategy; however, evidence suggests that many health care providers in Benue State were unaware of the PITC strategy. Benue State of Nigeria (at the time of this assessment) was one of the states with the highest HIV prevalence (5.0 – 10.6%) in the country, with variability in transmission rates in different Local Government Areas of the state [16]. Although the PITC strategy was implemented for pregnant women during antenatal care visits, this initiative was not broadened to include other adults who presented to the hospital as recommended.

Following the UNAIDS/WHO/CDC recommendation of PITC for HIV [9, 10], our health care center (General Hospital Idekpa) in Benue State, Nigeria recognised the need to increase HIV screening among adolescents and adults, and was the first in the state to incorporate routine provider-initiated HIV screening in all consultations for patients, including pregnant women. In this study, we aimed to assess the acceptability and logistical feasibility of the PITC strategy (under routine operational environment) among adolescents and adults who presented to the general out-patient clinic and antenatal care of General Hospital Idekpa in Benue State, Nigeria.

The implementation of the PITC approach in our local health facility was based on findings from previously published studies from Uganda [12], Botswana [17] and South Africa [18]. The PITC strategy was also implemented to provide context-specific evidence to determine the extent to which patients accepted HIV testing, and for public health practitioners to advocate for effective initiatives to improve the uptake of HIV testing in Benue State, Nigeria. Additionally, Nigeria is the largest recipient of developmental assistance for Health, including assistance for HIV prevention and control programmes [19]. Findings from this study will not only be of interest to public health agencies in Nigeria, but will also be of interest internationally to define the scope to which the PITC approach is context-specific.

## Methods

### Study setting

The study was implemented in a public health facility in Idekpa, Ohimini Local Government Area, Benue State of Nigeria. The Local Government Area has one comprehensive health care centre and seven primary health care centres with a population of 70,688 people based on the 2006 national census. In January 2010, the Benue State Government collaborated with the World Bank and Africa Development Bank to upgrade the comprehensive health centre in Idekpa to a secondary health care facility (General Hospital Idekpa), which is currently funded and managed by the Benue State Government. Before the upgrade, VCT was the standard approach to HIV detection, provided by a trained nurse and other lay health workers.

Approximately 900 patients visited the comprehensive health centre annually; of which, an estimated 70 patients (including pregnant women) were tested for HIV (8%), using the VCT approach. Following the upgrade of the comprehensive health centre to a General Hospital, an estimated 470 patients presented to the out-patient department and antenatal care unit within 6 months (June-December, 2010). Of these 212 patients (adolescents or adults aged ≥ 13 years) were eligible for the PITC strategy during this time interval, the period for this assessment. Patients (adults and children) who were HIV positive and taking anti-retroviral medications were not eligible. These patients were identified because they notified the attending clinical staff of their HIV positive status, and were counselled on the importance of drug compliance and follow-up care. In the six months following the implementation of the PITC strategy, an estimated 1,030 patients presented to the General Hospital. The increase in the latter was due to various community mobilisation activities implemented by the Benue State Government to create awareness among community members about the General Hospital upgrade.

In General Hospital Idekpa, health care services are provided at a cost (out-of-pocket), and are delivered by doctors, pharmacists, and trained nurses and midwives, including trained laboratory technicians. These service providers were trained in HIV/AIDS counselling and testing modalities. The PITC strategy for HIV screening was introduced in the health facility as a standard approach to health care, and counselling was provided by doctors trained nurses and midwives, consistent with the WHO/UNAIDS/CDC recommendation. At the start of the intervention in June 2010, the average patient’s turnout was 5–10 per week with adequate documentation of HIV testing and counselling.

### Implementation of the PITC strategy

The PITC strategy was implemented for all visiting eligible patients (adolescents or adults aged ≥ 13 years; *N* = 212) in the out-patient department and antenatal unit of General Hospital Idekpa, Benue State of Nigeria for the period (June–December, 2010). The implementation of this strategy aimed to assess the acceptability of HIV testing and counselling under normal operational and management conditions in the health facility to provide local data to relevant oragnisations. Random selection was not feasible because the strategy was aimed at all presenting patients. The implementation of the PITC strategy was facilitated by the health management team and first-line health professionals at the hospital. This approach made it possible to assess the logistical feasibility of the PITC strategy under routine operational environment of a secondary health care facility in a developing country.

The main outcome was the acceptability and logistical feasibility of routine provider-initiated HIV screening of new patients. The acceptability of the HIV test was measured as the proportion of patients who were offered HIV counselling and testing relative to those eligible for the test. Logistical feasibility was assessed as the proportion of patients who accepted HCT relative to those who were offered the test [12]. We also described the sample in terms of the patient’s gender, age, marital status and the proportion of those who have not being previously tested for HIV, including the test results, similar to previously published articles [12, 14].

### The provider initiated testing and counselling (PITC) strategy for HIV

The PITC strategy employed in this assessment was a modified version of the ‘ACTS’ approach, which includes four brief steps: assess, obtain consent, test, and provide supportive services [20]. In this study, a doctor or a trained nurse/midwife (trained in HIV testing and counselling) conducted the assessment to identify eligible participants, obtained verbal consent, and offered HIV testing as a standard part of medical care for all eligible patients, regardless of the patient’s presenting symptoms. The patients were allowed to decline or opt-out of the current testing. According to the WHO guideline for the provider-initiated HIV screening, verbal consent is allowed for the test to be performed [11]. Abbreviated pre-test counselling was also conducted, which consisted of informing patients about the HIV disease and that a person can only detect the disease by doing an HIV test; a recommendation that they should test for HIV at the index consultation; and patients would receive relevant services whether or not the test is positive or negative. If the person agreed, the doctor or the nurse/midwife would do a brief test readiness assessment, obtain verbal informed consent, and send the patient for a rapid HIV test along with other investigations that the patient may require. A patient refusal to take the test did not affect the quality of health service received, nor did it affect the patient-health provider relationship or confidentiality. Where a test offer was refused, further opportunities were created for the patient to either access a VCT or PITC in the future.

Rapid HIV testing was used, and the sequential (serial) rapid testing algorithm was employed where Determine HIV-1/2 assay kit (Abbott Laboratories, Illinois, United States of America) was used for screening; Uni-Gold test kit (Trinity Biotech, Wicklow, Ireland) was used as a confirmatory test, and HIV-1/2 STAT-PAK Dipstick assay (Chembio Diagnostic Systems Inc., New York, USA) was employed as the tie-breaker.

An HIV-negative result with the Determine assay was reported as negative. An HIV-positive result with the Determine assay was confirmed with the Uni-Gold test, and was reported as positive if both (Determine and Uni-Gold) tests gave positive results. If the Determine and Uni-Gold test results were discordant, the sample was subjected to a third test – the STAT-PAK assay. The result was reported as positive if the STAT-PAK assay result was positive, and negative if both Uni-Gold test and STAT-PAK assay results were negative.

Patients received post-test counseling and test results on the same day – within 60 min in most cases. Test results were provided to patients by the medical team (doctor or nurse/midwife). Health care providers encouraged patients to disclose their HIV status to their sexual partners and/or family members when they felt ready to do so. Additionally, health care providers assisted with disclosures if requested by the patient. The disclosures of HIV test results to either sexual partners or family members/friends were conducted by health professionals who had undergone appropriate training in HIV counselling. All patients, (whether HIV-negative or HIV-positive) were counselled on risk reduction after the test. Patients who tested HIV-positive were also provided information on available HIV/AIDS care and support, and co-trimoxazole (septrin) prophylaxis, and tuberculosis screening was initiated on diagnosis, consistent with the National HIV treatment guidelines. On discharge (if admitted) or after counselling, patients who tested HIV-positive were given referrals to HIV/AIDS specialised clinics (based on patient’s choice) for follow-up care.

### Data collection and analysis

Data collected from participants who were offered PITC included: age, gender, educational level, marital status, religion, history of HIV testing, most recent sexual partners and knowledge of HIV status of sexual partner (for those who reported sexual contact within the last six months prior to presentation at the hospital). Reasons for declining the current testing, and result of the HIV test (for those who accepted the test) were also documented. Frequencies and corresponding percentages with confidence intervals were calculated using Fisher's exact. All analyses were conducted in SPSS 2007 version 15.0.

### Ethics

Ethical approval for this assessment and subsequent publication of findings was obtained from the Benue State Ministry of Health Human Research Ethics Committee. All patients and caregivers (on behalf of children 13–17 years) were aware that as part of the clinical care, their demographic and clinical data were registered in paper-based clinic registers, stored in the hospital medical registry. Data employed for this assessment were anonymous, and the ethics committee did not require that the investigators sought written informed consent from patients based on the WHO PITC guideline. All patients or caregivers provided verbal informed consent to health care workers before they were allowed to participate.

## Results

The introduction of the PITC strategy (from June to December 2010) increased the number of patients who tested for HIV in the health facility compared to the VCT approach (i.e., from 8.0% in the 12 months prior to the upgrade of the health centre to 13.3% in the period of this assessment). The 8.0% was estimated based on the total number of patients seen in the comprehensive health centre in 12 months (900 patients) and the number of patients tested for HIV (70 patients) over the same period, using the VCT approach. The 13.3% was estimated based on the total number of patients seen at the General Hospital (1,500 patients) annually and 212 patients tested for HIV over the study period, using the PITC strategy. This proportion (13.3%) underestimates the actual impact of the PITC strategy because it was implemented for six months compared to the VCT which was conducted for one year. Of the 212 patients who were offered PITC for HIV, 199 (93.9%) accepted the HIV test, 10 (4.7%) opted out and 3 (1.4%) were undecided (Table 1). The reasons for opting out of the test included: being sure of their HIV status (*n* = 6); to inform husband/partner before taking the HIV test (*n* = 3) and not being ready for HIV testing (*n* = 1).

**Table 1:** Characteristics of the study population

Characteristics	N^a^ = 212 (%)	N^b^ = 199 (%)
Acceptability of HIV test		
Yes	199 (93.9)	
No	10 (4.7)	
Undecided	3 (1.4)	
Gender		
Female		131 (65.9)
Male		68 (34.1)
Age (years)		
13–20		29 (14.5)
21–30		52 (26.1)
31–40		43 (21.6)
41–50		35 (17.6)
51–60		20 (10.1)
60+		20 (10.1)
Marital status		
Single		119 (59.8)
Married		48 (24.1)
Separated/divorced		8 (4.0)
Widowed		24 (12.1)
Education		
No education		47 (23.6)
Primary education		67 (33.7)
Secondary education		65 (32.7)
Tertiary education		20 (10.0)
Religion		
Christian		192 (96.5)
Muslim		7 (3.5)
Previously tested for HIV		
Yes		57 (28.6)
No		142 (71.4)
Result of previous HIV test, if known		
Negative		47 (23.6)
Unknown		10 (5.0)
Most recent (last 6 months) sexual partner		
Husband		30 (15.1)
Steady partner		107 (53.8)
Casual partner		4 (2.0)
None		58 (29.1)
Result of HIV test		
Positive		18 (9.0)
Negative		181 (91.0)

N^a^: Total number of participants who were offered HIV testing

N^b^: Total number of participants who accepted HIV testing

More than half of the patients who accepted the HIV test reported a steady sexual partner in the six months preceding their presentation to the hospital (54%). Almost a quarter of patients reported being previously tested for HIV, and knew their HIV status (24%). Among the patients who tested for HIV, many tested negative (91%), while nine percent tested HIV positive (Table 2). The PITC strategy was logistically feasible in General Hospital Idekpa, Benue Sate of Nigeria because HIV testing was affordable and quick, and counselling was provided by health care professionals (doctors and nurses/midwives). These findings were compared to evidence from selected sub-Saharan African countries (Table 3).

**Table 2:** Prevalence of HIV cases in the population by study factor

Characteristics	HIV cases	(%) 95%(LCI-UCI)	*P* value
Gender			
Female	14	10.6 (5.9–17.2)	0.434
Male	4	5.8 (1.6–14.4)	
Age (years)			
13–20	1	0.3 (0.1–17.7)	0.430
21–30	5	9.6 (3.1- 21.0)	
31–40	5	11.6 (3.9–25.1)	
41–50	1	2.9 (0.1–14.9)	
51–60	4	20.0 (5.7- 43.7)	
60+	2	10.0 (1.2–31.7)	
Marital status			
Single	9	7.5 (3.5–13.9)	0.048
Married	2	4.6 (0.1–14.2)	
Separated/divorced	3	37.5 (8.5–75.5)	
Widowed	4	17.7 (4.7–37.3)	
Education			
No education	4	8.5 (2.7–20.4)	0.255
Primary education	10	14.9 (7.4–25.7)	
Secondary education	4	6.1 (1.7–15.0)	
Tertiary education	0	-	
Religion			
Christian	18	9.3 (5.7–14.4)	0.541
Muslim	0	-	

*LCI* Lower Confidence Interval, *UCI* Upper Confidence Interval

N: Total number of participants who accepted HIV testing

%: Proportions of HIV cases with 95% Confidence Intervals (CIs)

**Table 3:** Summary of provided initiated HIV testing and counselling implementation results from selected sub-Saharan African countries

Authors	Country	Study setting	N^a^	N^b^	% (LCI-UCI)	N^c^	% (LCI-UCI)	N^d^	% (LCI-UCI)
Kharsany et al., 2010 [26]	South Africa	Hospital-based	5612	2439	43.0 (42.0–45.0)	3173	57.0 (55.0–58.0)	1378	56.0 (55.0–58.0)
Wanyenze et al., 2008 [12]	Uganda	Hospital-based	51642	50649	98.0 (98.0–98.0)	993	2.0 (2.0–2.0)	12107	0.24 (24.0–24.0)
Leon et al., 2010 [14]	South Africa	Hospital-based	2326	1752	75.0 (74.0–77.0)	574	25.0 (23.0–26.0)	326	0.19 (17.0–21.0)
Bassett et al., 2007 [18]	South Africa	Hospital-based	2912	1414	49.0 (47.0–50.0)	1498	51.0 (50.0–53.0)	463	0.33 (30.0–35.0)
Ijadunola et al., 2011 [15]	Nigeria	University	252	251	99.6 (98.0–1.00)	1	40.0 (0.0–2.0)	0	0.00 (0.0–1.0)
Topp et at., 2011 [24]	Zambia	Hospital-based	41861	31197	75.0 (74.0–75.0)	10644	34.0 (34.0–35.0)	6572	0.21 (21.0–22.0)

*LCI* Lower Confidence Interval, *UCI* Upper Confidence Interval

N^a^: Number of test offered; N^b^: Number of test accepted; N^c^: Number of offered test declined; N^d^: Number of HIV positive

### Discussion of the study findings, with sub-Saharan African comparison

A well-coordinated routine PITC strategy that was integrated into the general health care services, and delivered by health care professionals in a secondary health facility in Benue State Nigeria was highly acceptable and logistically feasible, with majority of patients opting for HIV testing who previously did not know their HIV status. In comparing the 6-months period of the PITC implementation in our facility with the epoch (one year) pre-implementation using the VCT approach, the PITC strategy appears to be more effective in the hospital environment. Consistent with previous studies from Benue state Nigeria [21, 22], this study found a higher prevalence of HIV testing among participants who had primary/secondary education compared to those who had no schooling. In addition, the PITC strategy provided information on HIV transmission and prevention (during the pre- and post-test counseling) to the patients and the community. Our findings were consistent with those from other developing countries (such as South Africa, Uganda, Rwanda, Zambia and Botswana) with high prevalence of HIV. These studies also found that PITC was highly acceptable and feasible, and increased the proportion of patients who tested for HIV compared to the VCT approach [12, 17, 18, 23, 24].

The high acceptance of the PITC strategy for HIV testing, with an option to decline has been shown to reduce maternal-to-child transmission of HIV in populations [17, 25]. Consistent with a study from South Africa [26], our study found that the reasons as to why patients declined to take the HIV test included: being tested previously; the believe that they were not at risk; the need to obtain consent from their partner/husband before taking the HIV test; and not being ready for the test. Additional reasons for why patients may accept or refuse an HIV test using the PITC strategy include cost of the HIV test [12] or a lack of money [27]; level of referral system, access to HIV specialised care and skill of the health care provider [14]; human resources at the health facility [12]; and confidentiality [28].

Although patients paid for the HIV test at the facility (at a cost of one hundred and fifty naira, equivalent of one United Sates dollar – based on the 2010 exchange rate), the study found that a large proportion of patients accepted the HIV test compared to a study from South Africa [18]. The South African study reported that more than half of the patients declined to be tested (51%). This refusal may be due to the fee charged for the HIV test or a reflection of the patient’s right to decline the test – part of the ethical implication of the PITC strategy. The payment for the HIV testing (rapid test kits) was employed by the hospital management team because the implementation of a fully funded HIV program was still under consideration by relevant health and donor agencies at the time of the assessment. Despite this payment, a high acceptance rate was observed in this study, consistent with findings from Uganda which introduced the free PITC strategy [12]. The high acceptance rate in our facility was likely to be multifactorial. Firstly, patients were probably less apprehensive in participating in the PITC approach because the strategy may have been perceived by patients as a “standard of care” offered to all patients, thereby reducing the risk of stigma and other adverse social concerns attached to HIV. In many communities, stigma and discrimination continue to be the prevailing response to HIV/AIDS diagnosis, particularly in the VCT (opt-in) approach, probably because special HIV clinics are used for the VCT strategy [29]. Secondly, community sensitization and availability of the on-site rapid HIV testing may have played a significant role in the high acceptance rate and HIV testing among patients. In many sub-Saharan African countries (including Nigeria), health care professionals are well respected by the community and are seen as change agents. This perception may have played a role in this study as health professionals directly offered the HIV test to patients, similar to other studies from sub-Saharan Africa [12, 18, 23].

Findings from this study have operational connotations beyond the HIV testing and counselling strategy as it relates to the management of staffing resources. In this study, doctors and nurses integrated HIV screening into their medical consultations, taking on a duty previously performed by a less skilled worker or trained HIV counsellor. The strategy employed in this study is different from ‘task shifting’ recommended by the WHO, where responsibilities are referred down to junior health workers in order to reduce consultation times for treatment, care and support of HIV patients [30, 31]. The PITC approach capitalised on existing staffing resources, particularly in resource poor settings such as Nigeria because it was incorporated into routine medical consultations performed by skilled health professionals, consistent with previous studies [12, 14]. The expansion in doctors and nurses’ roles was achieved with meetings of the hospital management team and front-line clinical staff. This strategy extended consultation times by approximately ten to fifteen minutes on average, and was similar to a study from Rwanda [23]. However, in communities where trained health personnel (such as doctors and nurses) are limited, trained lay health workers who regularly consult patients can initiate the PITC strategy to increase HIV testing and coverage [32].

Policies and procedures to promote early detection of HIV infection in adolescents and adults using the routine PITC strategy have raised ethical concerns with regard to the definition and implementation of the strategy, particularly in settings with high proportion of illiteracy, poverty, gender disparities, poor access to treatment and fragile healthcare structure [33, 34]. Despite these concerns, the PITC is voluntary, and involves the “three Cs” – informed consent, counselling and confidentiality – that must be observed. In the opt-out approach, adolescents and adults must explicitly decline the HIV test after receiving the pre-test education. In contrast, the patient must consent to the HIV test after receiving the pre-test education in the opt-in strategy [35]. In comparing the VCT approach to PITC, a population-based survey from Botswana found that PITC reduced barriers to HIV testing, increased the proportion of people who tested for HIV and was broadly supported by the community [17]. Efforts to implement the PITC strategy must strictly consider and adhere to relevant guidelines with regard to protecting individual confidentiality, informed consent and ensuring adequate linkages to treatment, care and support [36].

Despite the introduction of the VCT and PITC approaches in the hospital environment to increase uptake of HIV testing, studies from developing contexts have shown that community-based HIV testing and counselling approaches (that is, testing outside of health facilities) had a higher coverage and uptake, and identified people living with HIV who were unware of their HIV status compared to hospital-based HIV testing strategies [37–39]. These findings highlight the need for a comprehensive strategy that involves facility- and community-based HIV testing approaches to effectively increase uptake of HIV counselling and testing in Nigeria's communities. Aspects of these initiatives can include a complete roll-out of the PITC strategy, mobile testing, door-to-door testing, self-testing, index testing, workplace testing, school-based testing, church-based testing and outreach testing, streamlined with local resources and galvanised with political support. Previous reports have revealed that these measures were effective in increasing the uptake of HIV testing [39, 40].

A number of challenges were encountered during the implementation of the opt-out strategy. Firstly, the clinics were understaffed with regard to nurses. In many settings, nurses are usually trained in HIV counselling to improve HIV testing rate; but while nurses in our facility were trained in HIV counselling, the increase in clinic workload due to staff shortages resulted in reluctance among nurses to take up additional duty of the PITC strategy. Studies on the experiences of health care workers in relation to the implementation of PITC strategy are warranted. Secondly, the VCT approach should have been assessed at the General Hospital because this information would have been very useful for effective comparison of both VCT and PITC approaches. Thirdly, studies from South Africa and Burkina Faso found lower prevalence of HIV testing among males [18, 41]; however, our assessment of the PITC strategy among males was challenging because fewer males compared to females presented to the facility. This male pattern of health seeking behaviour has been reported elsewhere [42–44]. Fourthly, the HIV prevalence observed in this study may not be representative of the overall HIV prevalence in the community because primary health centres in the local environment also provided HIV VCT services to patients. Despite these limitations, our study provides local evidence on the acceptability and logistical feasibility of the PITC strategy in Nigeria for informing HIV prevention and control initiatives. Additionally, all patients (severally ill or not) who presented to the out-patient department were included in this assessment, and this approach ensures early detection of HIV seropositive patients and subsequent referral for early treatment and care, when compared to a previously published study [18].

## Conclusion

The PITC strategy that was incorporated into the out-patient clinic and antenatal care unit of a secondary health care centre in Idekpa, Nigeria was highly acceptable and logistically feasible. The strategy also increased HIV testing rate among people who previously have not been tested for HIV (by 5%) compared to the VCT approach. Interventions and policy responses that facilitate health care centres to initiate the PITC strategy in Nigeria should be encouraged, but this approach must consider individual confidentiality, informed consent and counselling as well as the local context.

## Abbreviations

AIDS: Acquired immunodeficiency virus
CDC: Centre for disease control
HCT: HIV counselling and testing
HIV: Human immunodeficiency virus
PITC: Provided initiated testing and counselling
UNAIDS: Joint United Nations Programme on HIV/AIDS
VCT: Voluntary counselling and testing
WHO: World Health Oragnisation
